# Diffuse Cerebral Vasospasm After Aneurysmal Subarachnoid Hemorrhage in a 15-Year-Old Girl: A Case Report

**DOI:** 10.3389/fradi.2021.774739

**Published:** 2022-02-14

**Authors:** Dajeong Lee, Jeehun Lee, Keon Ha Kim, Ji Hye Kim, Jiwon Lee

**Affiliations:** ^1^Department of Pediatrics, Samsung Medical Center, Sungkyunkwan University School of Medicine, Seoul, South Korea; ^2^Department of Radiology, Samsung Medical Center, Sungkyunkwan University School of Medicine, Seoul, South Korea

**Keywords:** subarachnoid hemorrhage, diffuse cerebral vasospasm, seizure, pediatric multiple aneurysm, triple-H therapy

## Abstract

Diffuse cerebral vasospasm after subarachnoid hemorrhage (SAH) is a complication resulting in an ischemic condition presenting with altered mentality and followed by motor or speech impairment. It is uncommon in pediatric population and requires differential diagnosis from Moyamoya disease, which is relatively common in Korea. We report a case of a 15-year-old girl who was presented with a seizure and subsequent headache, poor oral intake, and altered mentality, who was finally diagnosed with sporadic vasospasm followed by multiple aneurysm ruptures. The patient had recurrent seizures and persistent headache at the time of transfer. On the second day after transfer, she showed focal motor weakness and dysarthria, and her symptoms gradually progressed, showing paraplegia and aphasia on the third hospitalization day. Brain magnetic resonance imaging and magnetic resonance angiography demonstrated diffuse narrowing cerebral vasospasm of bilateral middle cerebral arteries, anterior cerebral arteries, and distal internal carotid arteries and three unruptured aneurysms. The patient was treated with intravenous hydration and nimodipine to expand the narrowed vessels. After confirming that the vessels were enlarged, we successfully executed the endovascular coil embolization. Her neurological deficits were improved through medical, interventional, and rehabilitation treatments and fully restored 11 months after discharge.

## Introduction

Cerebral vasospasm is a prolonged but reversible narrowing of the cerebral arteries occurring several days after subarachnoid hemorrhage (SAH). It is associated with the rupture of cerebral aneurysms within the basal cisterns in 50–90% of cases (1–3). It occurs in 3–5 days after aneurysmal rupture, appears to be the narrowest diameter of the blood vessel in 5–14 days, and resolves slowly within 2–4 weeks (3–5). The clinical symptoms begin with altered mentality and accompanying motor or speech impairment (3, 5). It is confirmed by cerebral angiograms and has been reported to occur in 70% of SAH cases (4–6).

In the pediatric population, symptomatic vasospasm is unusual, and its exact prevalence is elusive. We describe the case of a 15-year-old girl who presented with recurrent seizures, persistent headache, and sudden-onset motor weakness subsequently, who was finally diagnosed with sporadic vasospasm caused by multiple aneurysmal ruptures.

## Case Report

A previously healthy 15-year-old girl was transferred to the pediatric neurology department with recurrent seizures and persistent headache lasting for 10 days. The first seizure occurred 11 days before the transfer to this hospital. It was a focal seizure with impaired awareness lasting for 5 min. After the seizure she complained of general weakness, headache, and vomiting. She was afebrile, and her neurological examination showed neck stiffness. Her brain magnetic resonance imaging (MRI) showed no abnormal findings, and electroencephalography showed intermittent sharp wave discharges from the right posterior head region. Cerebrospinal fluid (CSF) analysis revealed a red blood cell count of 85,000/mm^3^, white blood cell count of 70/mm^3^, protein concentration of 76.8 mg/dL, and glucose concentration of 53 mg/dL. When the pleocytosis in CSF analysis was corrected in consideration of the count of red blood cell in CSF, it was calculated as a negative value. Nevertheless, she was treated with intravenous ceftriaxone because that finding alone could completely rule out central nervous system infection. All microbiological examinations performed on CSF specimens were confirmed to be negative. After 3 days, her body temperature rose to 38°C. Upon admission, she complained of persistent headache, which made her bedridden. Her headaches and fever got better thereafter. On the eighth hospitalization day, she experienced the second generalized tonic seizure. Then she became drowsy or stuporous and her electroencephalography showed slowing of the posterior head region. The thyroid autoantibodies were found to elevate: free T4 1.99 ng/dL, anti-microsomal antibody (Ab) 657.1 IU/mL, and anti-thyroglobulin Ab 1,164 IU/mL (normal range: 0.80–1.99 ng/dL, <16 IU/mL, and <100 IU/mL, respectively). Intravenous methylprednisolone (1 g/day) was administered for 5 days under the impression of Hashimoto's encephalopathy based on these findings.

**Figure 1:**
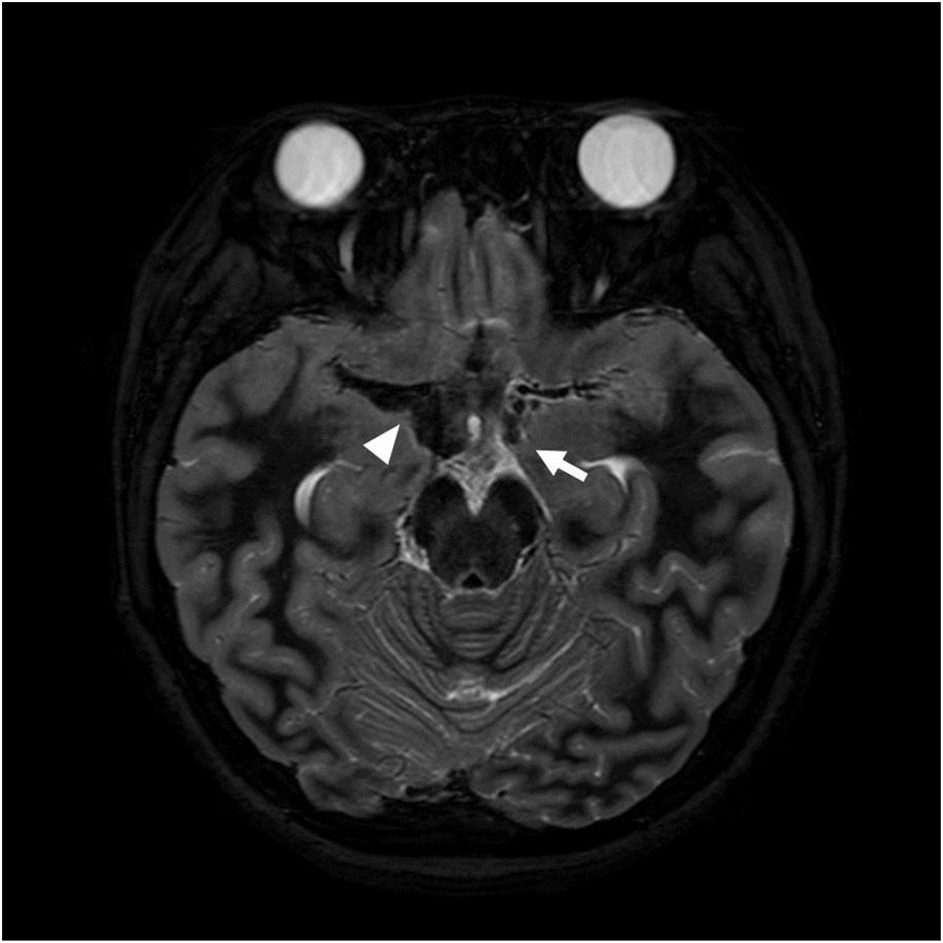
Axial T2 weighted image obtained on the day of transfer shows low signal intensity (arrowhead) adjacent to the right middle cerebral artery (MCA) and an aneurysm at the left distal ICA (arrow) suggesting subarachnoid hemorrhage resulted from aneurysmal rupture.

**Figure 2:**
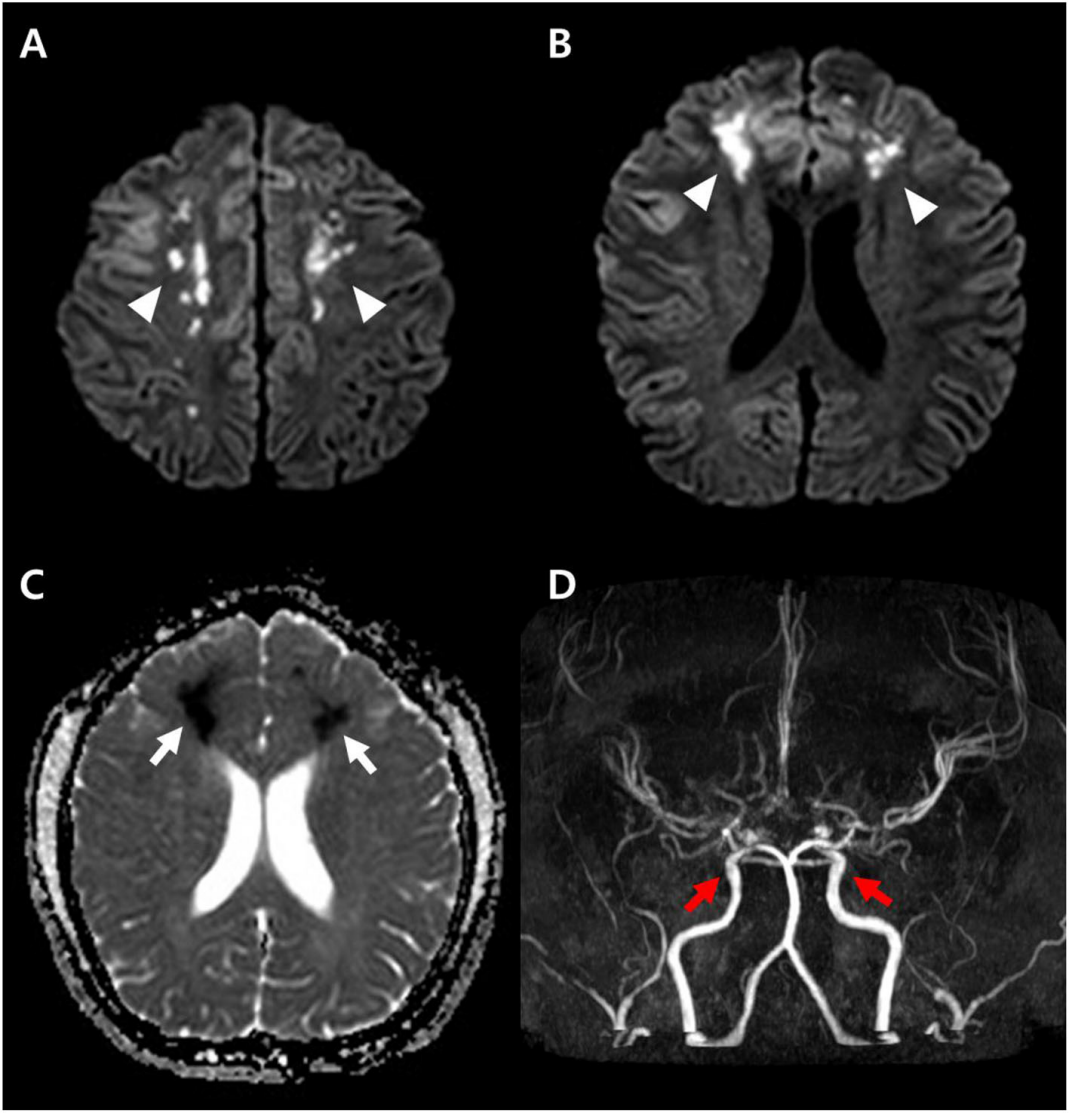
Diffusion weighted image obtained at the time of onset **(A,B)** shows high signal intensity lesions at both parasagittal areas (arrowheads), and apparent diffusion coefficient (ADC) map **(C)** shows low signal intensity (arrows) suggesting acute cytotoxic edema. The brain magnetic resonance angiography (MRA) demonstrates diffuse narrowing of the cerebral arteries and small aneurysms (red arrows) at both distal internal carotid arteries **(D)**.

**Figure 3:**
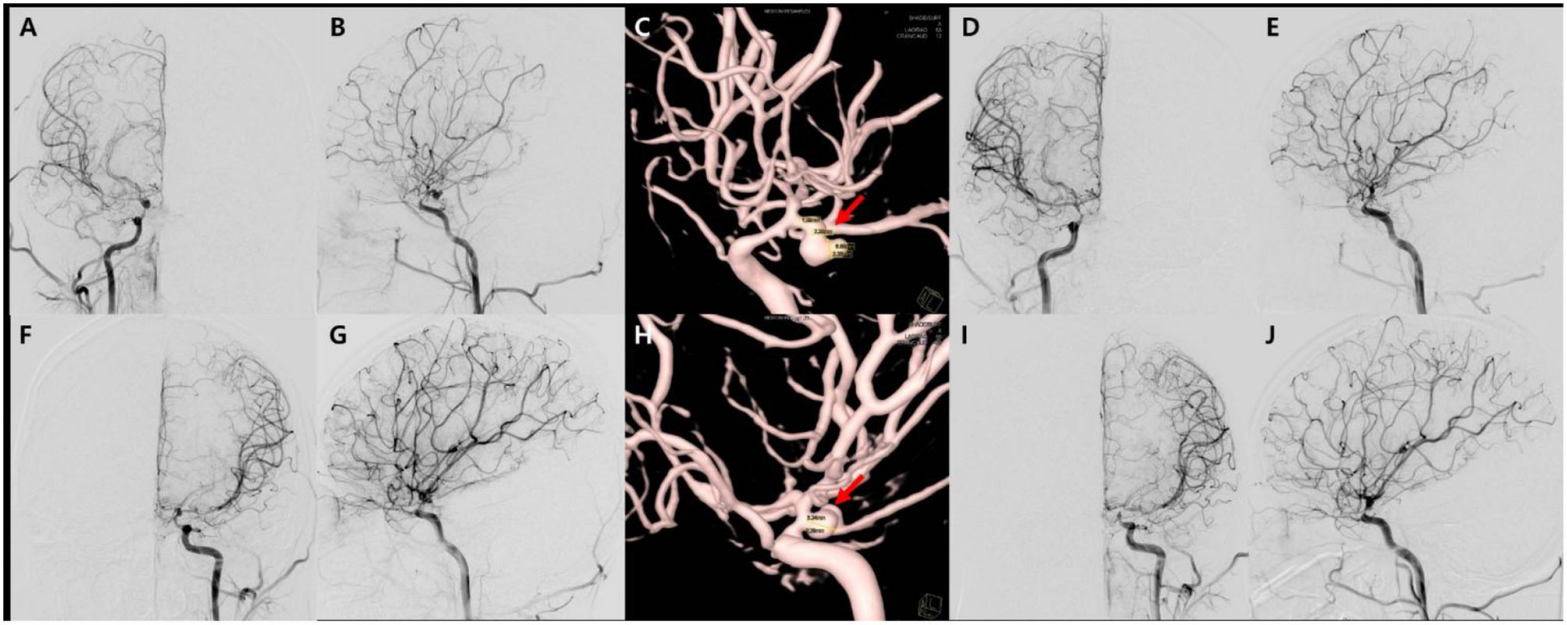
Transfemoral cerebro-arteriography and subsequent coiling of the aneurysms. There are diffuse narrowing of both distal internal carotid arteries, middle cerebral arteries, and anterior cerebral arteries, as well as and multiple unruptured aneurysms in both anterior choroidal artery and the left middle cerebral artery on angiography in anteroposterior **(A,F)**, lateral **(B,G)** views, which is better demonstrated on 3D reconstruction images **(C,H)**. The first endovascular coil embolization for aneurysms on the right anterior choroidal artery on anteroposterior **(D)** and sagittal views **(E)**, respectively. Secondary coiling was performed on left anterior choroidal artery aneurysm in the angiography on anteroposterior **(I)** and sagittal views **(J)**.

**Figure 4:**
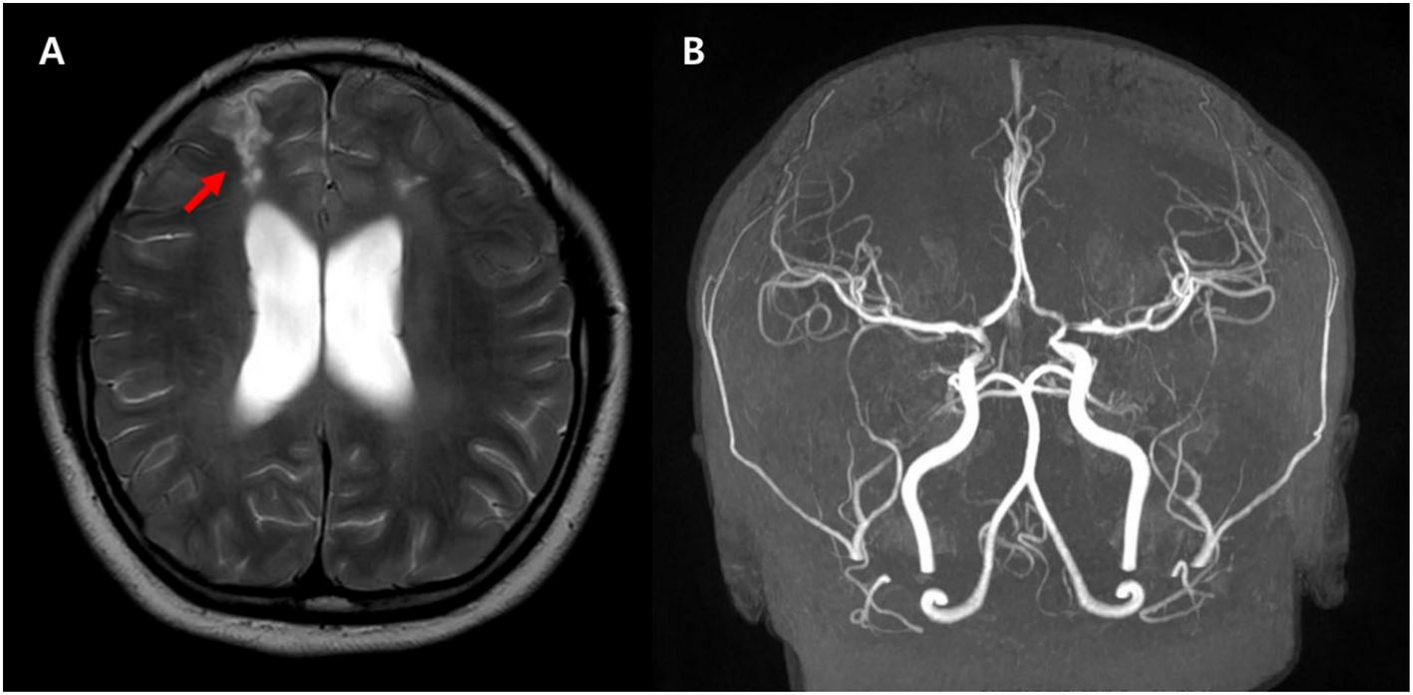
Follow-up axial T2 weighted images **(A)** demonstrate the high signal intensity in both parasagittal areas suggesting the watershed injury (arrows). The MRA **(B)** shows improved narrowing of both MCAs and ACAs suggesting improved diffuse spasm of the arteries. There are remaining multifocal stenosis of the vessels at both distal internal carotid arteries, proximal middle cerebral arteries, and proximal anterior cerebral arteries.

She was transferred to Samsung Medical Center with continuous alteration of mental status after the second seizure on the 12th day of hospitalization. On admission to this hospital, she was drowsy and sometimes confused. The patient consistently complained of severe headache. On the second hospitalization day, sudden weakness affecting both legs and dysarthria appeared. The symptoms waxed and waned during the day. On the third hospitalization day, her symptoms progressed into paraplegia and aphasia. Upon neurological examination, the Babinski sign and ankle clonus were positive bilaterally. The brain MRI on the day of transfer showed acute hemorrhage around the distal internal carotid artery (ICA) and right middle cerebral artery (MCA) obliterating the basal cistern and narrowing of both distal ICA and aneurysm at the left distal ICA (Figure 1). The brain MRI and magnetic resonance angiography (MRA) on the third hospital day demonstrated diffuse narrowing of bilateral MCAs, anterior cerebral arteries (ACAs), and distal ICAs, as well as small aneurysms at both distal ICAs (Figure 2D). In addition to the persistent SAH in the basal cistern and prepontine cistern, multifocal parasagittal acute infarctions were found (Figures 2A–C). Those findings raised the possibility of diffuse vasospasm associated with the SAH resulted from aneurysm rupture. The vessel wall MRI showed no contrast enhancement in the arterial wall, which could exclude the cerebral angiitis. Transfemoral cerebral angiography (TFCA) was performed on the sixth hospitalization day, revealing three unruptured aneurysms in both anterior choroidal arteries and left MCA and posthemorrhagic vasospasm involving both distal ICAs, MCAs, and ACAs (Figures 3A,F). The degree of narrowing of these cerebral blood vessels was severe for considering the endovascular coil embolization of the aneurysms. Therefore, we administered nimodipine and provided sufficient intravenous hydration to expand the vessels. On the seventh hospitalization day, after confirming that the vessels were widened enough to perform the intervention, we executed the first endovascular coil embolization for obliteration of unruptured aneurysms on the right anterior choroidal artery (Figures 3B–E). The upper motor neuron sign disappeared after the procedure. On the ninth hospitalization day, she became alert; however, the motor aphasia continued. She began to speak in sentences on the 15th day of hospitalization. On the seventeenth hospitalization day, we performed the second endovascular coil embolization for obliterating the left anterior choroidal artery aneurysm (Figures 3G–J). Although her brain MRI showed a slight reduction of infarction size compared to the previous results after the embolization, her clinical symptoms improved with the rehabilitation treatment. She was discharged on the forty-third hospitalization day without any motor deficit. The brain MRI and MRA performed 10 months after discharge demonstrated encephalomalacia in both parasagittal areas especially in the left frontal lobe (Figure 4). The electroencephalogram performed at that time showed epileptiform discharges in the left or right frontal areas. On her last follow-up in the outpatient clinic 14 months after discharge, her motor function was normal, and no neurologic deficit was observed.

## Discussion

This is a pediatric case of diffuse cerebral vasospasm resulting from the SAH associated with aneurysmal rupture. The patient presented with a seizure, followed by severe headache, fever, and neck stiffness, which was almost resolved in a week. The initial brain MRI was unremarkable, and the EEG revealed focal epileptiform discharges. The patient had another seizure in a week and became drowsy and confused with motor weakness and dysarthria. A follow-up brain MRI with MRA revealed diffuse cerebral vasospasm and diffusion restriction in the border zone. The presence of aneurysms and SAH on follow-up MRI could explain the cause of diffuse vasospasms and the various symptoms of the patient. The treatment of the aneurysm solved her neurologic symptoms.

The most common cause of diffuse cerebral vasospasm in the pediatric population is SAH (7–10). Sixty percent of children with SAH have cerebral aneurysms. The affected area of the brain is deprived of oxygen-rich blood, resulting in stroke and vasospasm. Vasospasm usually occurs 5–10 days after SAH and reduces cerebral blood flow, causing a secondary stroke (3, 4, 11, 12). When SAH occurs, the patient may have symptoms of sudden-onset severe headache, nausea, blurred or double vision, loss of consciousness, seizures, and neck stiffness, which also occurred in the patient of this report. The conventional TFCA is the gold standard for identifying vasospastic vessels (4, 13), and it also provided the cause of vasospasm in this case.

Moyamoya disease can be considered when diffuse arterial narrowing is found on brain MRA and would be an important differential diagnosis, especially in northeastern Asia. It is an occlusive cerebrovascular disease characterized by stenosis of the supraclinoid branches of the ICA (14, 15). It causes the formation of an abnormal vascular network at the base of the brain. Most children with Moyamoya disease present with episodic ischemic symptoms, especially during hyperventilation. Conversely, adults show ischemic complications in half and intracranial hemorrhage in the other half (14–16). SAH associated with Moyamoya disease has been reported to have a low incidence and is extremely rare in pediatric populations. The differentiating factors between diffuse cerebral vasospasm after SAH and Moyamoya disease are waxing and waning neurologic deficit, presence of Moyamoya vessels on MRA, and existence of distal arterial branches, which are consistent findings for Moyamoya disease (3, 15). The patient in this case study did not show any episodic ischemic symptoms previously, and angiography findings showed diffuse vasospasm without the presence of distal cerebral arteries or Moyamoya vessels.

Reversible cerebral vasoconstriction syndrome (RCVS) can be a differential diagnosis in this case. RCVS can present with thunderclap headache, with or without focal neurologic deficits and/or seizures (17, 18). The differential point is that the angiography of RCVS is typically “strings and beads” appearance that is different from the homogenous vasospasm found in this case.

The treatment for correcting vasospasm includes the use of hypervolemic, hypertensive, and hemodilutional therapy, also known as triple-H therapy. Medical treatments such as aggressive intravenous volume expansion and administration of inotropic agents can optimize cerebral perfusion and restore impaired neurological deficits (3, 4, 19). When extracellular calcium enters the cell, vascular contraction continues, and nimodipine, a calcium channel blocker, can be used as a vascular relaxant (3, 4, 19). Percutaneous transluminal angioplasty can be considered as a treatment for vasospasm; it was not possible in our case because it was only applicable to proximal vasospasm. For the remaining multiple aneurysms, we performed endovascular coiling several times to prevent additional rupture.

## Conclusion

This case demonstrates a rare pediatric vasospasm associated with SAH after aneurysm rupture. The presenting symptom was generalized seizure followed by long-lasting severe headache and neck stiffness. The rarity of diffuse cerebral vasospasm in pediatric patients makes clinicians differentiate several possible diagnoses such as Moyamoya disease, RCVS, and hemiplegic migraine, as well as vasospasm associated with SAH.

## Data Availability Statement

The original contributions presented in the study are included in the article/supplementary material, further inquiries can be directed to the corresponding author/s.

## Ethics Statement

The studies involving human participants were reviewed and approved by Samsung Medical Center. Written informed consent to participate in this study was provided by the participants' legal guardian/next of kin.

## Author Contributions

DL, JeL, and JiL contributed to conception and design of the study. KK who performed the TFCA and coiling treatment assisted in selecting intervention images. JK helped with the description and selection of brain MRI and MRA images. DL wrote the first draft of the manuscript. JeL and JiL corrected the manuscript. All authors contributed to manuscript revision, read, and approved the submitted version.

## Conflict of Interest

The authors declare that the research was conducted in the absence of any commercial or financial relationships that could be construed as a potential conflict of interest.

## Publisher's Note

All claims expressed in this article are solely those of the authors and do not necessarily represent those of their affiliated organizations, or those of the publisher, the editors and the reviewers. Any product that may be evaluated in this article, or claim that may be made by its manufacturer, is not guaranteed or endorsed by the publisher.
